# Identification of novel *STAT5B* mutations and characterization of TCRβ signatures in CD4+ T-cell large granular lymphocyte leukemia

**DOI:** 10.1038/s41408-022-00630-8

**Published:** 2022-02-24

**Authors:** Dipabarna Bhattacharya, Antonella Teramo, Vanessa Rebecca Gasparini, Jani Huuhtanen, Daehong Kim, Jason Theodoropoulos, Gianluca Schiavoni, Gregorio Barilà, Cristina Vicenzetto, Giulia Calabretto, Monica Facco, Toru Kawakami, Hideyuki Nakazawa, Brunangelo Falini, Enrico Tiacci, Fumihiro Ishida, Gianpietro Semenzato, Tiina Kelkka, Renato Zambello, Satu Mustjoki

**Affiliations:** 1 Hematology Research Unit Helsinki, University of Helsinki and Helsinki University Hospital Comprehensive Cancer Center, Helsinki, Finland; 2 Translational Immunology Research Program and Department of Clinical Chemistry and Hematology, University of Helsinki, Helsinki, Finland; 3 Department of Medicine, Hematology and Clinical Immunology Branch, University of Padova and Veneto Institute of Molecular Medicine (VIMM), Padova, Italy; 4 Department of Computer Science, Aalto University, Espoo, Finland; 5 Institute of Hematology and Center for Hemato-Oncology Research, University and Hospital of Perugia, Perugia, Italy; 6 Department of Internal Medicine, Division of Hematology, Shinshu University School of Medicine, Matsumoto, Japan; 7 Department of Biomedical Laboratory Sciences, Shinshu University School of Medicine, Matsumoto, Japan; 8 iCAN Digital Precision Cancer Medicine Flagship, Helsinki, Finland

**Keywords:** Chronic lymphocytic leukaemia, Cancer genetics, T-cell receptor

## Abstract

CD4+ T-cell large granular lymphocyte leukemia (T-LGLL) is a rare subtype of T-LGLL with unknown etiology. In this study, we molecularly characterized a cohort of patients (*n* = 35) by studying their T-cell receptor (TCR) repertoire and the presence of somatic *STAT5B* mutations. In addition to the previously described gain-of-function mutations (N642H, Y665F, Q706L, S715F), we discovered six novel *STAT5B* mutations (Q220H, E433K, T628S, P658R, P702A, and V712E). Multiple *STAT5B* mutations were present in 22% (5/23) of *STAT5B* mutated CD4+ T-LGLL cases, either coexisting in one clone or in distinct clones. Patients with *STAT5B* mutations had increased lymphocyte and LGL counts when compared to *STAT5B* wild-type patients. TCRβ sequencing showed that, in addition to large LGL expansions, non-leukemic T cell repertoires were more clonal in CD4+ T-LGLL compared to healthy. Interestingly, 25% (15/59) of CD4+ T-LGLL clonotypes were found, albeit in much lower frequencies, in the non-leukemic CD4+ T cell repertoires of the CD4+ T-LGLL patients. Additionally, we further confirmed the previously reported clonal dominance of TRBV6-expressing clones in CD4+ T-LGLL. In conclusion, CD4+ T-LGLL patients have a typical TCR and mutation profile suggestive of aberrant antigen response underlying the disease.

## Introduction

T-cell large granular lymphocyte leukemia (T-LGLL) is a rare lymphoproliferative disease characterized by chronic expansion of clonal, mature cytotoxic T cells in the peripheral blood and bone marrow [1]. Two subtypes of T-LGLL are commonly recognized: the most common CD8+ T-LGLL (70% of cases) and the less frequent CD4+ T-LGLL (30% of cases) [1]. Clinically, CD4+ T-LGLL is usually an indolent disease and unlike in its CD8+ counterpart, these patients rarely have cytopenias or autoimmune symptoms. However, CD4+ T-LGLL has been reported more frequently associated with secondary neoplasms such as monoclonal B-cell lymphocytosis and plasma cell disorders [2, 3]. In CD4+ T-LGLL, the leukemic T-LGLs express CD4 (either alone or together with CD8) and the α/β T-cell receptor (TCR) together with a typical mature cytotoxic (Granzyme B+, CD56+, CD57+) and activated/memory T-cell (CD2^bright^, CD7^dim^, CD11a^bright^, CD28−, CD62L−) phenotype [3].

Up to 55% of CD4+ T-LGLL patients have been shown to harbor *STAT5B* mutations [4, 5]. In CD8+ T-LGLL, the most common mutated gene is *STAT3* [6], whereas *STAT5B* mutations are rare and often associated with an aggressive disease form [7–9]. All reported *STAT5B* mutations in CD4+ T-LGLL are point mutations within the SH2 or transactivation domains of *STAT5B*. N642H and Y665F are the most common *STAT5B* mutations, and they both have been shown to increase STAT5B protein activity [4, 10, 11].

The etiology of CD4+ T-LGLL remains unknown. An initial antigen-driven expansion of CD4+ T cells, followed by the occurrence of oncogenic events (i.e., somatic mutations), has been suggested to lead to the persistence of abnormal T-cell clones [11]. Non-self-antigen(s) instead of autoantigens [5] are proposed targets of CD4+ T-LGL clones as CD4+ T-LGLL is not associated with autoimmune diseases. In some earlier reports, CD4+ T-LGL clones have been implied to recognize cytomegalovirus (CMV) antigens [12, 13]. In CD4+ T-LGLL [3], the enrichment of the Vβ13.1 gene usage has also been reported, differentiating it from CD8+ T-LGLL where no enrichment of specific Vβ gene usage has been observed [14, 15]. Moreover, CD4+ T-LGLL patients with a monoclonal expansion of TCRVβ13.1 display a common HLA-DRB1*07:01 genotype and are reported to display an identical motif (QG) in the middle of the CDR3 sequence [16, 17]. These observations suggest that the evolution of the expanded CD4+ T-LGLL clones is not a stochastic process, but rather a result of an antigen-driven immune response [18–22].

As CD4+ T-LGLL is a rare disease, previous studies evaluating the clinical impact of *STAT5B* mutations have been limited in size, and no deep TCR profiling has been performed with modern sequencing and bioinformatic tools [23–25]. Therefore, we aimed to collect a large cohort of CD4+ T-LGLL patients (*n* = 35) and examine by deep amplicon sequencing the *STAT5B* mutation status and correlate the genotype information with the clinical data. Additionally, by deep TCRβ sequencing, we studied the landscape of both the leukemic and non-leukemic T-cell repertoires in CD4+ T-LGLL and compared that to the landscapes from the healthy controls and patients with CD8+ T-LGLL.

## Materials and methods

### Patient cohort

CD4+ T-LGLL patients (*n* = 35) were recruited from the hematology units of 3 academic institutions, namely Padova (Italy; *n* = 22 patients), Helsinki (Finland; *n* = 6 patients) and Shinshu (Japan; *n* = 7 patients). Buffy coats from healthy blood donors (*n* = 37) were provided by the Finnish Red Cross Blood service (Helsinki, Finland). The study was conducted in accordance with the Ethics Committees (Padova University Hospital ethics committee, approval number 4213/AO/17; Helsinki University Hospital ethics committee, approval number 303/12/03/01/2011; Shinshu University Hospital ethics committee, approval number 581). All patients gave written informed consent prior to their inclusion in the study in accordance with the Declaration of Helsinki.

### Sample preparation

Peripheral blood mononuclear cells (PBMC) were isolated with Ficoll-Hypaque (Sigma Aldrich) gradient centrifugation. Leukemic clones were purified using magnetic micro-beads coated with monoclonal anti-human CD4, CD57, or CD56 antibodies (Miltenyi Biotec). When available, cryopreserved cells (*n* = 15) were first subjected to depletion of CD8+ cells, after which the CD8-negative population (or CD8+ in case of CD4+ CD8+ double-positive LGL clone) was further purified with anti-human CD4-conjugated microbeads. Alternatively, CD57 (*n* = 8) or CD56 (*n* = 3) purified LGLs or PBMC (*n* = 9) were used depending on sample availability. A similar approach was used to purify CD4+ cells from buffy coats obtained from healthy controls (*n* = 37). Flow cytometry analysis was conducted to control the purity of the obtained cell fractions (>95% among lymphocytes) with FACSCanto II in Padova and FACS Verse in Helsinki; data were analyzed with FACS Diva and FACS Suite software, respectively (all Becton Dickinson). Surface staining was performed according to the purification: CD4 FITC CD8 PE or CD4 FITC CD57 PE or CD4 FITC CD56 APC (all Becton Dickinson). DNA was extracted using Gentra Puregene Cell and Tissue kit (Qiagen) and quantified using the Qubit 2.0 fluorometer with DNA high sensitivity kit (ThermoFisher Scientific).

### Amplicon sequencing of *STAT5B*

**Fig. 1:**
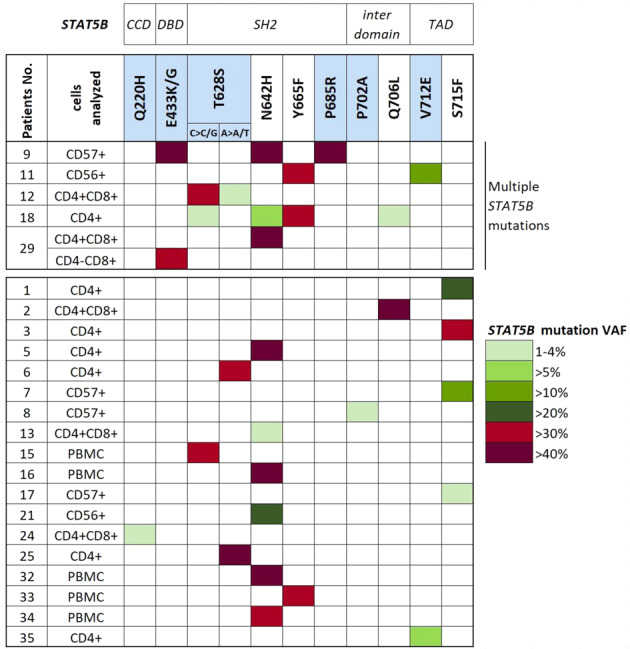
*STAT5B* mutations and variant allele frequencies in CD4+ T-LGLL. *STAT5B* mutations and the corresponding VAFs were evaluated in the LGLs or PBMC populations by deep amplicon sequencing. Novel *STAT5B* mutations are in light blue squares. The patients are divided into those with multiple *STAT5B* mutations (above) and with single *STAT5B* mutation (below). The square colors indicate mutation VAF percentages, as reported in the legend. The protein level localization of the identified *STAT5B* mutations is given on the top row. *CCD* coiled coil domain, *DBD* DNA-binding domain, *TAD* transactivation domain, *VAF* variant allele frequency.

*STAT5B* sequencing was performed from LGL or PBMC fraction (Fig. 1) to detect somatic mutations. Firstly, targeted sequencing was performed starting from 50 ng of DNA. Libraries, generated according to the manufacturer’s instructions, were sequenced on an Illumina MiSeq instrument using MiSeq Reagent kit v2 or v3 and following the TruSeq Custom Amplicon Assay (Illumina) pipeline. Bioinformatic variant calling was performed using MiSeq Reporter software (version 2.5.1) with default settings while for variant annotation Illumina Variant Studio 2.0 was used. Next, the observed mutations were confirmed using amplicon sequencing as previously described in Kim et al. [26]. Amplicon primers used for both techniques are reported in Supplementary Table 1. Germline variants were excluded by examination of a non-leukemic cell population from each patient. All identified variants were visually confirmed using the Integrative Genome Viewer (IGV) [27].

### Western blot assay and *STAT5B* luciferase reporter assay

Please refer to Supplementary methods and Supplementary Table 2.

### TCRβ sequencing and data analysis

TCRβ sequencing from genomic DNA (the same DNA as used for amplicon sequencing, Fig. 1 and Table 1) of CD4+ T-LGLL patients (*n* = 27) was conducted with Adaptive Biotechnologies’ ImmunoSEQ assay with “Survey” resolution [28]. Only productive, i.e., complete, in-frame, TCRβ rearrangements were included in the analyses. Sample clonality was assessed using the Simpson clonality as provided by the Adaptive Immunoseq Analyzer (ver 3.0). Healthy controls (*n* = 785) from peripheral blood [29] were also used after downsampling to 40,000 reads.

**Table 1:**
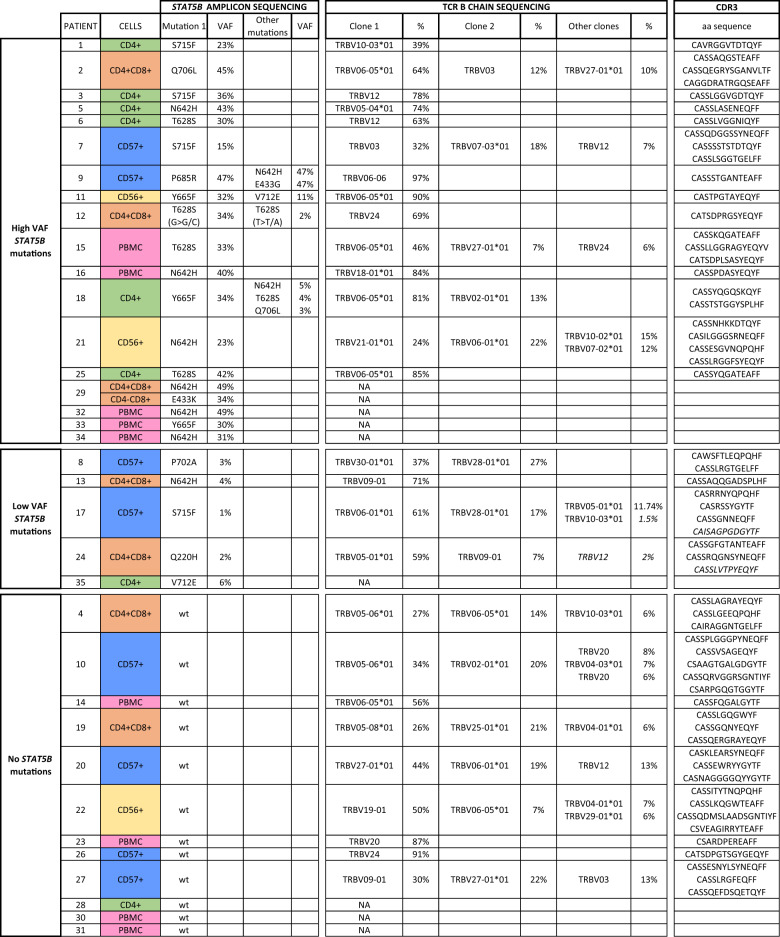
*STAT5B* mutations and TCRβ clonotypes in CD4+ T-LGLL patients.

*STAT5B* mutation status, type of the *STAT5B* mutation and TCR Vβ chain sequences (>5%) with their relative percentage (%) are given for each patient. For patients #17 and #24, two additional clones <5% are reported considering the putative match with the low *STAT5B* mutation VAF (in italics). The CDR3 amino acid sequences are listed according to the % of the Vβ expansions (from the highest to the lowest). Color codes the cell of origin used in the sequencing: green, CD4+ sorted cells; orange, CD4+CD8+ sorted cells; blue, CD57+ sorted LGLs; yellow, CD56+ sorted LGLs; pink, PBMC.

*VAF* variant allele frequency, *wt* wild-type, *PBMC* peripheral blood mononuclear cells.

Clones, defined as unique rearranged receptor sequences in the immunoSEQ Analyzer, were classified as very rare (≤0.01%), small (0.01–0.1%), medium (0.1–1%), large (1–20%), and hyperexpanded (>20%). The lowest detectable productive frequency in the TCR-sequencing data was 2.59 × 10^−5^. OLGA [25] (ver 1.2.3) was used to calculate the generation probabilities of the TCR rearrangements with default parameters.

VDJtools was used to generate circus plots for V gene usage from TCRβ-sequencing data [30]. The query against the VDJ database (VDJdb; ver 2021-01-10) [24] was done for all available TCRs from CD4+ cells. To evaluate possible amino acid level similarities of TCRs, GLIPH (ver 1.0.0 and 2.0.0) [23, 31] was used on a remote server with default parameters.

### Statistical methods

Statistical analysis for the evaluation of differential *STAT5B* transcriptional activities through luciferase reporter assay was performed with a two-way ANOVA followed by Dunn’s multiple correction test. Fisher’s exact, Chi-square, or Bonferroni corrected Mann–Whitney tests were used to analyze the relationship between *STAT5B* mutational status, TCRβ repertoire covariates, and relative clinical features of the cohort of patients profiled (see Table 2 for more details). Fisher’s exact test with Bonferroni multiple correction was used to calculate V genes enrichment for CD4+ T-LGLL clones. All the analyses were performed with GraphPad Prism 6 (ver 6.07) or R (ver 4.0.2) and *p* < 0.05 was considered significant.

**Table 2:** Clinical features of the CD4+ T-LGLL cohort.

	a	b		
	CD4 + LGLL *n* = 35	*STAT5B* mutated *n* = 23	*STAT5B* wt *n* = 12	*p*-value
Females (%)	17 (49%)	8 (35%)	9 (75%)	**0.035**
Males (%)	18 (51%)	15 (65%)	3 (25%)	
Median age (range)	72 (41–86)	74 (42–86)	69 (41–86)	0.23
Median WBC, ×10^9^/l (range)	9.4 (1.4–20.9)	10.5 (1.4–20.9)	6.9 (3.4-11.4)	**0.006**
LGL count, ×10^9^/l (range)	3.45 (0.43–8.86)	4.31 (0.43–8.86)	2.03 (0.43–6.10)	**0.006**
CD4+CD8− (%)	17 (49%)	11 (48%)	6 (50%)	0.48
CD4+CD8+ (%)	11 (31%)	9 (39%)	2 (17%)	
Both expansions (%)*	7 (20%)	4 (17%)	3 (25%)	
CD57+CD56+CD16+ (%)	4 (12%)	4 (17%)	0 (0%)	0.32
CD57+CD56+CD16− (%)	26 (79%)	16 (70%)	10 (83%)	
CD57+CD56−CD16− (%)	3 (9%)	1 (5%)	2 (17%)	
ANC, ×10^9^/l (range)	2.73 (0.6–7.31)	3.06 (0.71–7.31)	2.22 (0.6–3.88)	0.07
Hb, g/l (range)	140 (123–163)	146 (126–163)	136 (123–150)	0.06
PLT, ×10^9^/l (range)	222 (139–330)	222 (139–330)	223 (163–298)	>0.99

Clinical information was collected at diagnosis. Data (**a**) from the entire cohort of CD4+ T-LGLL patients and data from the same patients (**b**) split by the presence of somatic *STAT5B* mutations. For two *STAT5B*-mutated patients no CD57, CD56, and CD16 expression data was available. Two *STAT5B* mutated and one wild-type patients presented a double leukemic expansion (CD57+CD56 ± CD16−) and were associated to CD57+CD56+CD16− group since it was the prevalent clone. Statistically significant *p*-values (*p* < 0.05) between the *STAT5B* mutated and wild-type patients are highlighted in bold. CD4/CD8 and CD57/CD56/CD16 immunophenotype distributions between the *STAT5B* mutated and wild-type patients were analyzed using the Chi-square test, whereas sex prevalence was analyzed with Fisher’s exact test. All other clinical features were analyzed with Mann–Whitney *U*-test.

*WBC* white blood cells, *LGL* large granular lymphocytes, *ANC* absolute neutrophil count, *Hb* hemoglobin, *PLT* platelets, *wt* wild-type.

^*^The patients present a double expansion, in six cases the main clone is CD4+CD8− and the minor one is CD4+CD8+ whereas only one case presents a major clone CD4+CD8− and a minor one CD4−CD8+.

## Results

### *STAT5B* mutation status is associated with clinical features in CD4+ T-LGLL

Clinical characteristics of the entire CD4+ T-LGLL cohort (*n* = 35) are shown in Table 2a. The median age at diagnosis was 72 years (ranging from 41 to 86 years) and no gender bias was observed (ratio Males/Females = 1.06). White blood cell (WBC) and LGL counts were overall high (9.4 ± 4 × 10^9^/l and 3.45 ± 2.40 × 10^9^/l, respectively). Absolute neutrophil count (ANC), hemoglobin (Hb), and platelets (PLT) were mostly within the normal range. Half of the patients (49%, *n* = 17) presented a CD4+CD8− leukemic expansion while 31% (*n* = 11) had a CD4+CD8+ leukemic clone. Moreover, 20% (*n* = 7) of the patients harbored two coexisting clonal expansions, six presenting one CD4+CD8− and one CD4+CD8+ clone, whereas patient #29 presented one CD4+CD8+ and one CD4−CD8+ clone (Supplementary Table 3). All CD4+ T-LGLL clones were CD57+ and the majority of them (79%, *n* = 26 patients) were CD56+CD16−. Three patients harbored an expanded CD56−CD16− clone. Conversely, none of the patients presented a CD56−CD16+ T-LGLL clone. A detailed table of the clones and associated clinical features is presented in Supplementary Table 3.

**Fig. 2:**
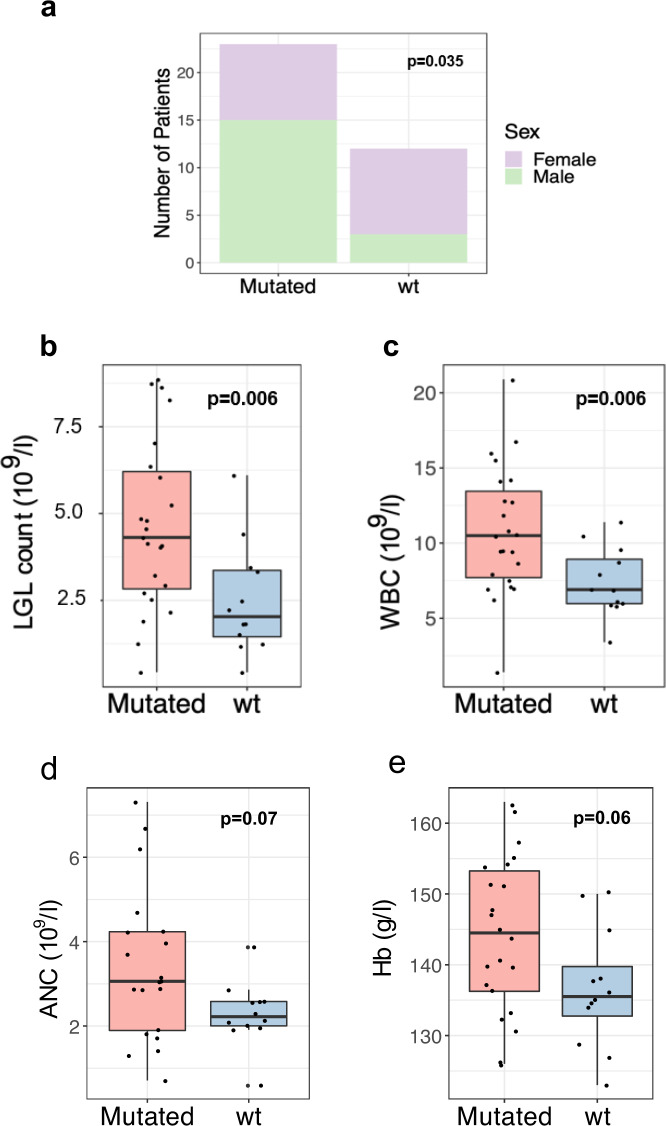
Most relevant clinical features associated with *STAT5B* mutations. Box and bar plots of the clinical features in *STAT5B* mutated and wild-type patients: **a** Sex, **b** LGL count, **c** WBC, **d** ANC, **e** Hb All clinical features were analyzed with Mann–Whitney *U*-test except for sex prevalence which was analyzed with Fisher’s exact test. *WBC* white blood cells, *LGL* large granular lymphocytes, *ANC* absolute neutrophil count, *Hb* hemoglobin, *wt* wild-type.

By targeted amplicon sequencing, we discovered that 23 out of 35 patients (66%) harbored somatic *STAT5B* point mutations (Fig. 1). No differences were detected in the median age between the two groups, whereas *STAT5B* mutations were more frequent in males (Fisher’s exact test, *p* = 0.035; Fig. 2a and Table 2b). Patients with *STAT5B* mutations displayed increased LGL counts and lymphocytosis when compared to the *STAT5B* wild-type patients (Mann–Whitney test, *p* = 0.006 for both variables; Fig. 2b, c and Table 1b). There was no statistically significant difference in the platelet counts between the mutated and wild-type patients, while a tendency towards higher ANC and Hb levels in the *STAT5B* mutated patients was noted (*p* = 0.07 and *p* = 0.06, respectively; Fig. 2d, e).

*STAT5B* mutations were particularly common in patients with CD4+CD8+ phenotype (82%, 9 of 11 patients). Furthermore, all patients (*n* = 4) having CD57+CD56+CD16+ phenotype had *STAT5B* mutation, whereas in patients with CD57+CD56+CD16− phenotype mutation prevalence was 62% (16 of 26 patients).

### Identification of multiple and novel *STAT5B* mutations

Among the mutated cases (*n* = 23), 78% of the patients (*n* = 18) harbored *STAT5B* mutations with a variant allele frequency (VAF) over 15%, whereas the remaining 22% of cases (*n* = 5) presented lower VAFs ranging from 1% to 6% (Fig. 1 and Table 1).

Multiple *STAT5B* mutations were detected in five patients (#9, #11, #12, #18, #29) (Fig. 1), corresponding to 22% of the mutated CD4+ T-LGLL cases. Careful examination of the sequencing reads revealed that in two cases (#12 and #18) the closely located genetic lesions were present in different alleles. For patient #29, amplicon sequencing was performed independently in the two purified leukemic clones. In the CD4+CD8+ clone, we identified the N642H variant (VAF 49%) and in the CD4−CD8+ clone, we identified the E433K mutation (VAF 34%). Patient #9 harbored P685R, N642H, and E433G mutations with almost identical VAF (47%), thus suggesting that these heterozygous mutations co-localized in the dominant leukemic clone (97%). In patient #11, the 2 mutations (Y665F, V712E) were sequenced in separate amplicons and although the exact clonal structure was not possible to establish, significantly varying VAFs (32% and 11%) suggest clonal heterogeneity.

Besides the previously identified *STAT5B* mutations in CD4+ T-LGLL (N642H, Y665F, Q706L, and S715F [4]), we discovered additional six novel somatic *STAT5B* mutations in the cohort. T628S (*n* = 5) and P685R (*n* = 1) mutations are located in the SH2 domain, whereas the V712E mutation (*n* = 2) is in the transactivation domain (TAD), both of which are mutational hotspot regions of the *STAT5B* gene. In addition, we identified four additional mutations that are located in the coiled-coil domain (CCD; Q220H, *n* = 1), DNA binding domain (DBD; E433G/K, *n* = 2), and in the inter-domain region (P702A, *n* = 1).

The most frequent mutation was N642H (9/23, 39%), followed by T628S (5/23, 22%), S715F (4/23, 17%) and Y665F (3/23, 13%). Two different base substitutions led to the same missense T628S mutation: the wild-type nucleotide triplet ACC was found to mutate to either AGC or TCC, both inducing the conversion from tyrosine (T) to serine (S). The first nucleotide alteration has already been listed in COSMIC [32], whereas the latter is novel. Interestingly, the two possible substitutions leading to the same amino acid conversion were both identified in different alleles (i.e., in different reads) of patient #12.

### E433K and V712E are novel activating *STAT5B* mutations associated with CD4+ T-LGLL

**Fig. 3:**
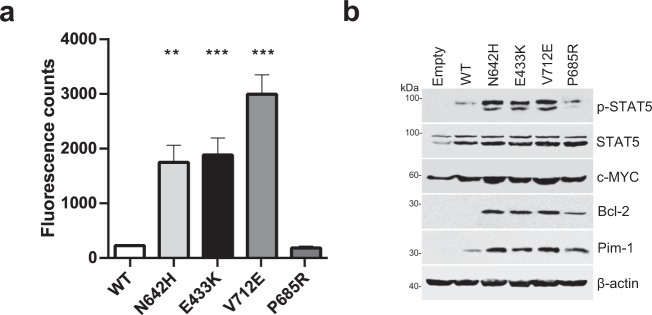
Functional characterization of the novel *STAT5B* mutations. **a** Luciferase reporter assay revealed the *STAT5B* transcriptional activity induced by the E433K, V712E, and P685R mutations. By using *STAT5B* mutants-transfected HeLa cells, luciferase activities of *STAT5B* mutations were compared to the wt *STAT5B* data and are reported as luminescence counts. WT *STAT5B*: a negative control*. STAT5B* N642H mutation: a positive control. Statistical analysis was performed using two-way ANOVA. WT: wild-type; **p < 0.001; ***p < 0.0001. **b** Protein expression in HeLa cells transiently carrying *STAT5B* mutants. The cells were serum-starved for 6 h after the transfection. Protein extracts were subjected to western blot assay with p-STAT5, STAT5, c-MYC, Bcl-2, and Pim-1 specific antibodies. β-actin served as loading control. Data are representative of the three independent experiments.

Functional characterization of E433K, V712E, and P685R mutations was performed to determine their impact on the transcriptional activity of the *STAT5B* gene; known activating variant N642H and the wild-type *STAT5B* constructs were used as positive and negative controls, respectively (Fig. 3a). Q220H and P702A mutations were excluded from the analysis because of their low VAF (2% and 3%, respectively). T628S mutation with gain-of-function property has already been described before in T-prolymphocytic leukemia (T-PLL) [33].

As shown in Fig. 3a, both E433K and V712E were found to be highly activating when compared to wild-type *STAT5B* (*p* < 0.001, two-way ANOVA followed by Dunn’s multiple correction test). There was at least a 10-fold increase in the luminescent signal for the E433K mutation and a 15-fold increase for the V712E mutation when compared to the wild-type *STAT5B*. The signal from the P685R mutated plasmid did not differ from the wild-type construct. This mutation was detected in the patient with co-existing N642H and E433K mutations. In addition, HeLa cells transiently carrying the *STAT5B* GOF mutants exhibited upregulated phosphorylation of STAT5B protein compared to the cells carrying WT *STAT5B* (Fig. 3b). Furthermore, *STAT5B* mutated cells had increased protein expression levels of c-MYC, Bcl-2, and Pim-1 which are downstream targets of STAT5 [34].

### Both the leukemic and non-leukemic TCRβ repertoires are highly clonal in CD4+ T-LGLL

**Fig. 4:**
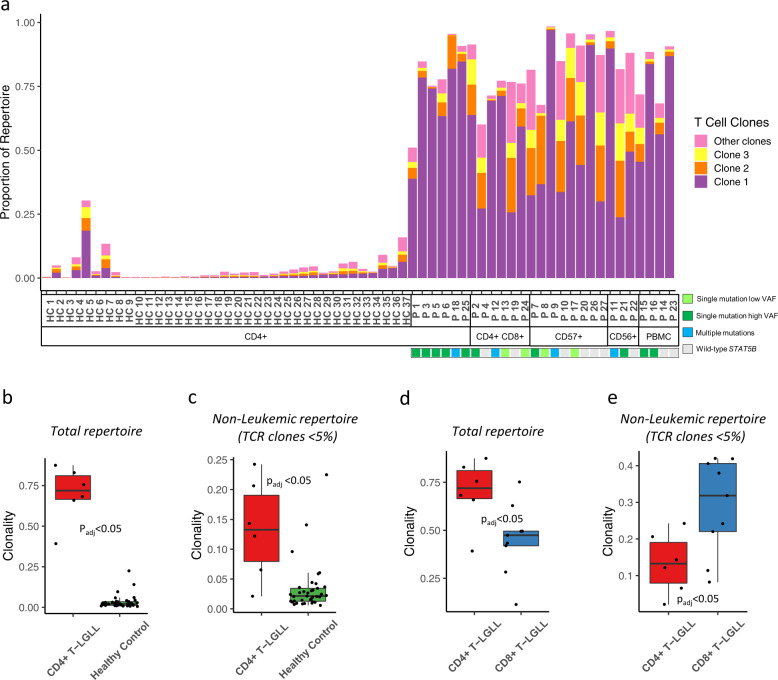
Clonal expansions in CD4+ T-LGLL and comparison of the TCRβ repertoires between the CD4+ T-LGLL and healthy samples. **a** The ten most expanded clones as proportion of total repertoire from all samples are shown. The top 3 clones are shown in individual colors while the sum of the rest (clones 4–10) is shown in one color. The cell isolation method (CD4, CD4 CD8, CD57, CD56, or non-selected PBMC) of the analyzed cell population and the mutational *STAT5B* status of the patients are given below the sample numbers. **b** Total CD4+ repertoire clonality as normalized Shannon–Wiener clonality index from CD4+ T-LGLL (patient samples purified for CD4, *n* = 6) and CD4+ cells from healthy controls (*n* = 37). **c** Non-leukemic CD4+ repertoire clonality (selecting the CD4+ T-LGL clones with a size < 5%) as normalized Shannon–Wiener clonality index from CD4+ T-LGLL (patient samples purified for CD4, *n* = 6) and CD4+ cells from healthy controls (*n* = 37). **d** Total repertoire clonality as normalized Shannon–Wiener clonality index from CD4+ T-LGLL (patient samples purified for CD4, *n* = 6) and CD8+ cells from CD8+ T-LGLL (patient samples purified for CD8, *n* = 9). **e** Non-leukemic repertoire clonality (selecting the CD4+ T-LGL clones with a size < 5%) as normalized Shannon–Wiener clonality index from CD4+ T-LGLL (patient samples purified for CD4, *n* = 6) and CD8+ cells from CD8+ T-LGLL (patient samples purified for CD8, *n* = 9). All *p*-values were calculated with a Mann–Whitney test and corrected with Bonferroni.

TCRβ sequencing (TCRβ-seq) was performed on samples from CD4+ T-LGLL patients (*n* = 27) and healthy controls (*n* = 37). From T-LGLL patients, genomic DNA was isolated from bead-separated cells (CD4+, CD4+CD8+, CD56+ or CD57+) or PMBC depending on sample availability (Table 1). In healthy controls, CD4+ selected fractions were used. As expected, CD4+ T-LGLL patients harbored larger T-cell clones than in healthy controls (Fig. 4a). Both monoclonal and oligoclonal expansion patterns were observed. By focusing on clones with clone size exceeding 5% of the repertoire, we identified a total of 59 possible T-LGLL clones from the 27 patients (Supplementary Table 4).

For the CD4+ sorted samples, the overall TCRβ clonality was higher in CD4+ T-LGLL (*n* = 6) as compared to healthy controls (*n* = 37) (Fig. 4b, *p*_adj_ < 0.05, Bonferroni corrected Mann–Whitney test). Even after removing the leukemic T-LGLL clones from the patient samples, the observed difference in the clonality persisted (Fig. 4c, *p*_adj_ < 0.05, Bonferroni corrected Mann–Whitney test). Thus, the non-leukemic TCR repertoires in CD4+ T-LGLL patients are less diverse than the CD4+ TCR repertoires in the healthy controls. By repeating the analysis with varying thresholds of 3–7% for selecting the putative T-LGLL clones, we showed that this was invariant to our chosen threshold of 5% (Supplementary Fig. 1).

We also compared a similarly profiled CD8+ T-LGLL cohort [35] (*n* = 9) with the CD4+ T-LGLL cohort. Surprisingly, the overall clonality in CD4+ T-LGLL samples was higher than in the CD8+ T-LGLL samples (Fig. 4d, *p*_adj_ < 0.05, Bonferroni corrected Mann–Whitney test), although it is known that CD8+ TCR repertoires are more clonal than CD4+ repertoires [36]. This was however not seen in the non-leukemic repertoires, where the non-leukemic compartments from CD8+ T-LGLL samples had higher clonality than the non-leukemic compartments from CD4+ T-LGLL samples (Fig. 4e, *p*_adj_ < 0.05, Bonferroni corrected Mann–Whitney test).

### Most CD4+ T-LGLL-associated *STAT5B* mutations are clonally restricted

For most of the patients (71%) presenting with a *STAT5B* mutated clone over 15%, the VAF of the *STAT5B* mutation corresponded to the size of the main T-LGLL clone: the mutation VAFs were half of the size of the TCR clones suggesting heterozygous mutations (Table 1). Only in patients #2 and #15, the mutation VAFs were slightly higher, suggesting that there was more than one T-cell clone harboring the same mutation. In patient #21 the mutation VAF (23%) matched with the combined size of the two main T-LGLL clones (24% and 22%), suggesting that the mutation is present either in one clone in homozygous/hemizygous fashion or in both clones as heterozygous (Table 1). Interestingly, patients with low VAF (<5%) *STAT5B* mutations (#8, #13, #17, and #24) had nevertheless large T-LGLL clones (37–71%) suggesting that they had either subclonal *STAT5B* mutations or the mutations were located outside the main clone (Table 1).

In patients with multiple *STAT5B* mutations, the clonal structure was evaluated based on the VAF and TCR clone size. In patient #9, the three mutations co-existed in the same clone based on their similar VAFs (47%) matching with the size of the main clone (97%) (Table 1). In patient #18, the Y665F (VAF 34%) and N642H (VAF 5%) mutations were detected in different subclones, and accordingly, also two expanded TCR clonotypes were detected (Table 1). Patient #11 had one large main TCR clone (90%) but two different *STAT5B* mutations with different VAFs (Y665F: 32% and V712E: 11%) (Fig. 1 and Table 1). This suggests possible clonal evolution and the presence of different genetic lesions in individual cells bearing the same TCR.

### CD4+ T-LGLL clonotypes are predominantly restricted to individual patients

**Fig. 5:**
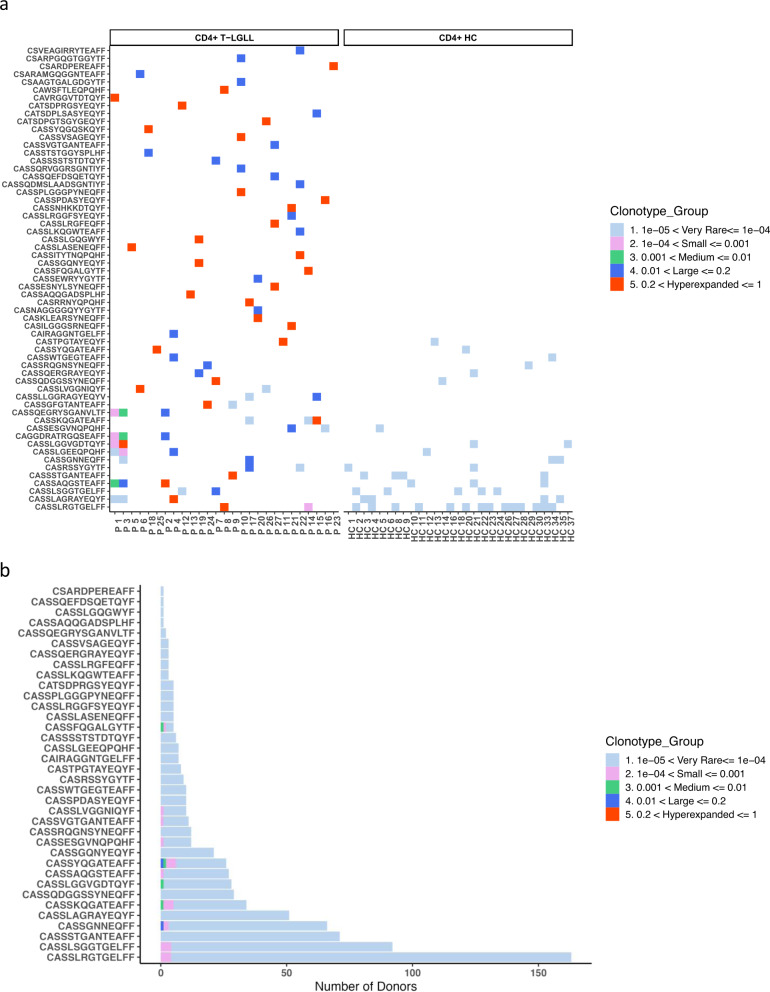
Publicness of the CD4+ T-LGLL clones. **a** CD4+ T-LGLL clonotypes are shared between CD4+ T-LGLL patients (*n* = 27) and healthy cohort (*n* = 37) in extremely rare frequencies. Shared clonotypes are at the bottommost rows and the colors refer to the size of the clonotype. **b** CD4+ T-LGLL clonotypes found in smaller frequencies in publicly available, unsorted, healthy cohort data (*n* = 786) [29] where the colors refer to the size of the clonotype.

To understand whether the 59 identified CD4+ T-LGLL clonotypes (Supplementary Table 4) could be a result of convergent evolution, we examined if these clones were shared between patients. In concordance with what has been reported before in CD8+ T-LGLL [14], none of the patients had the same TCR rearrangement in their main leukemic CD4+ T-LGLL clonotypes (Fig. 5a). However, 25% (15/59) of T-LGLL clonotypes were found in the non-leukemic TCR repertoires of other patients, albeit in smaller frequencies (ranging between frequencies of 1.41 × 10^−5^–1.27 × 10^−2^, Supplementary Table 5, Fig. 5a). Of these 59 clones, 27% (16/59) were found in healthy CD4+ sorted TCR repertoire (*n* = 37), again in much smaller frequencies (ranging between 8.24 × 10^−5^–2.59 × 10^−5^) (Supplementary Table 5, Fig. 5a). We further looked for the presence of the CD4+ T-LGLL clonotypes in a larger publicly available cohort (*n* = 786) of healthy donors’ unsorted TCR repertoire from Emerson et al. [29]. We observed that 36/59 of the patient clones (around 61%) were found at least once in the healthy donors with the most common clone being found in 163 healthy donors (Fig. 5b). However, most of these clones were found in extremely rare frequencies (ranging between 8.2 × 10^−5^ and 1.05 × 10^−5^).

We performed a similar analysis with our CD8+ T-LGLL cohort. None of the CD8+ leukemic clones (defined similarly as clones above 5%) was shared between the patients (Supplementary Fig. 2a). 50% of these clones were found in the Emerson et al. [29] cohort in low frequencies (Supplementary Fig. 2b).

Next, we calculated the generation probabilities of the leukemic TCRs with OLGA [25] as high generation probabilities could indicate a bias in the recombination and low generation probabilities towards convergent evolution. The TCR generation probabilities were higher in clones that were shared between healthy individuals. Conversely, the generation probabilities in clones that were exclusive to CD4+ T-LGLL were low (Supplementary Fig. 3).

### Skewed Vβ gene usage in CD4+ T-LGLL clones

**Fig. 6:**
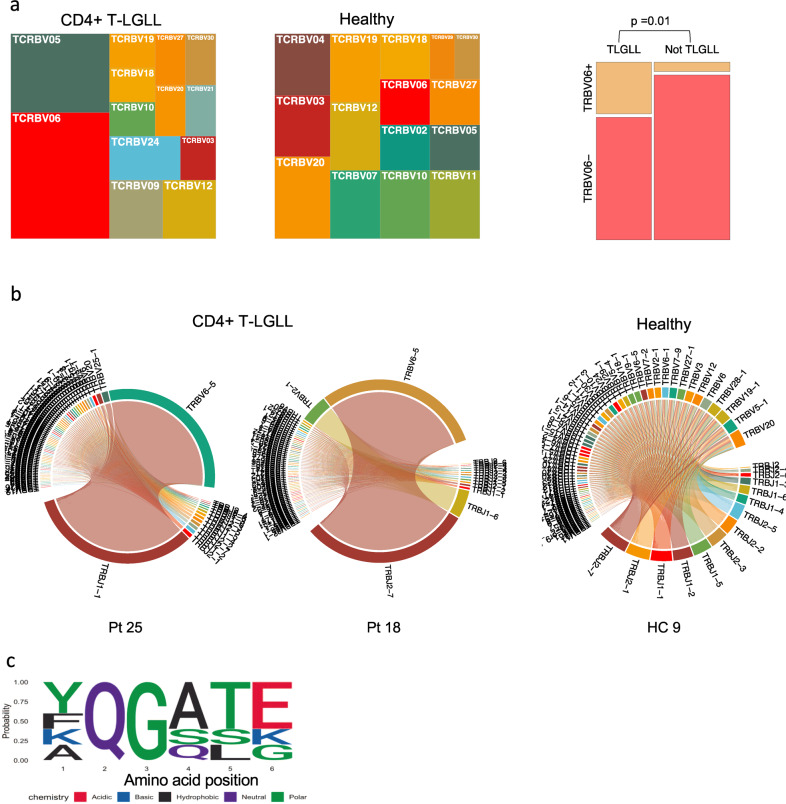
Structural similarities in CD4+ T-LGLL clones. **a** Comparison of V gene family usage between CD4+ T-LGLL clones and healthy controls. 20% of the leukemic clones express the *TRBV06* gene. **b** Circos plots showing skewed V gene usage in two representative CD4+ T-LGLL as compared to one representative healthy sample. **c** Logo plot showing the non-conservative middle part of TCRs CDR3, which is usually within 5 Å of the antigen. The “QG” motif that was found in the middle CDR3 in multiple CD4+ T-LGLL clones is shown in CDR3s that are 13 amino acids long.

Previous studies using Vβ flow cytometry analysis have suggested a biased usage of the Vβ13.1 family (consisting of *TRBV06-05*, *TRBV06-06*, and *TRBV06-09* genes) in CD4+ T-LGLL [16]. In our data, the predominant V-gene of CD4+ T-LGLL clonotypes belonged to the TRBV06 family in 20% (12/59) of CD4+ T-LGLL clonotypes (Supplementary Table 6, Supplementary Fig. 4). By selecting the single most expanded clones, we found that 8 out of 27 T-LGLL clones belonged to the *TRBV06* family, in comparison to only 2 out of the 35 clones belonging to the *TRBV06* family in healthy (Fig. 6a, *p* = 0.01, Fisher’s two-sided exact test). Representative examples from CD4+ T-LGLL, with both monoclonal and polyclonal expansions, and healthy are shown in Fig. 6b.

To test whether the CD4+ T-LGLL clones target a known viral antigen (from e.g., *CMV*, *EBV*, or *Influenza A*), we queried the 59 T-LGLL clones against the largest database containing known antigen specificities (VDJdb [24]) but no exact matches for TCRs recognizing epitope bound to class II HLAs were found. The antigen specificities of the non-leukemic TCRs returned multiple matches, with *Influenza A* as the most common target (Supplementary Fig. 5a).

Since structurally similar TCRs are known to recognize similar antigens [23, 31, 37], we used GLIPH to identify significantly enriched motifs within CD4+ T-LGLL clonotypes in comparison to healthy naïve repertories. Interestingly, we identified two conserved motifs within the 59 T-LGLL clonotypes. The first one, “SDP”, in three T-LGLL clones all of which shared the *TRBV24* gene, and the second one, “SLRG”, in three other T-LGLL clones with different V genes (Supplementary Table 7). No correlation was observed between motifs and the mutational statuses of the patients. Next, we analyzed whether the T-LGLL clonotypes shared motifs within their repertoire, i.e., whether the T-LGLL responses could be polyclonal antigen-specific responses. In 33% (2/6) of CD4+ sorted patients’ samples, we could identify a motif that was shared between leukemic and non-leukemic repertoires. Structurally similar TCRs from patient #18 are shown in Supplementary Fig. 5b, where the leukemic clone can be seen to share similarities with the multiple non-leukemic TCRs.

Although GLIPH does not consider motifs less than three amino acids long, Garrido et al. have previously reported the presence of the “QG” motif in the middle of the CDR3 region in patients with monoclonal Vβ13.1 expansion and HLA-DRB1*07:01 genotype [16]. We found this “QG” motif in 18.6% (11/59) of T-LGLL clones (of which only two expressed Vβ13.1), but also in 13.4% (20/144) of top 5 expanded clonotypes in healthy CD4+ repertoire (Fig. 6c, Supplementary Table 4, *p* = 0.39, Fisher’s two-sided exact test). To address whether “QG” is specific to CD4+ T-LGLL or specific to HLA-DRB1*07:01 in general, we analyzed whether “QG” was more common in CD4+ clones of HLA-DRB1*07:01 positive (*n* = 5) than negative (*n* = 32) healthy donors. We found that “QG” was enriched in HLA-DRB1*07:01 positive healthy donors (3.5% of CD4+ clones) in comparison to negative donors (0.54% of CD4+ clones; *p* = 7.53 × 10^−122^, Fisher’s one-sided exact test), further suggesting its association with CD4+ cells and HLA-DRB1*07:01 in general and not specifically to CD4+ T-LGLL.

## Discussion

Somatic *STAT5B* mutations were initially described in CD8+ T-LGLL [9], but follow-up analyses have revealed that they more often occur in CD4+ and immature T-cell malignancies [4, 33, 38–42]. Here, we performed a detailed molecular characterization of 35 CD4+ T-LGLL patients, which is the largest cohort described so far. We identified novel gain of function *STAT5B* mutations and found that leukemic clonotypes are predominantly private to the patients.

At least one *STAT5B* mutation was detected in 66% of the 35 CD4+ T-LGLL patients. This is the highest frequency of *STAT5B* mutations thus far reported in the literature. While the previously published studies have mostly focused on the mutational hotspot regions of *STAT5B* [5, 9], our sequencing assay covered the whole protein-coding sequence of *STAT5B*. Moreover, the use of a highly sensitive method allowed us to detect low-frequency mutations down to 1% VAF.

Functional analyses of the newly discovered *STAT5B* mutations confirmed STAT5 pathway activation, as the downstream targets of STAT5 (c-MYC, Bcl-2, and Pim-1 proteins) [34] were overexpressed. Similarly, a previous study has shown that *STAT5B* mutant-transduced cell lines upregulate Bcl-2 [43]. c-MYC is an essential regulator of cell proliferation and survival [34], and Pim-1, an anti-apoptotic gene, is known to synergize with c-MYC in leukemogenesis [44]. Altogether, this data suggests that the *STAT5B* GOF mutations are likely to be involved in clonal dominance and maintenance, probably by conferring proliferation advantage for the mutated cells. Accordingly, *STAT5B* mutated CD4+ T-LGLL patients had higher lymphocyte and LGL counts compared to wild-type patients.

In addition to the earlier described N642H, S715F, and Y665F mutations, we discovered the T628S mutation to be common in CD4+ T-LGLL (22% of our cohort). This mutation has been previously reported as a highly activating mutation in T-PLL [33, 45] and hepatosplenic T-cell lymphoma (HSTCL) [42]. Although SH2 and TAD domains are the known mutation hotspot area in the *STAT5B* gene, we also identified novel variants in the CCD (Q220H) and DBD (E433K/G) of *STAT5B*. Similarly, the majority of the *STAT3* mutations are in the SH2 domain in CD8+ T-LGLL but additional mutations are also found in other domains [4] underlining the importance of covering all mutation hotspots in the diagnostic tests of LGLL.

Among the *STAT5B* mutated CD4+ T-LGLL patients, 22% harbored multiple *STAT5B* mutations either in the main T-LGLL clone or in separate smaller clones. Interestingly, mutation VAFs did not always correlate with the T-cell clone size based on TCRβ sequencing, suggesting sub-clonal heterogeneity. Similarly, 17% of CD8+ T-LGLL patients have been reported to harbor multiple *STAT3* mutations [46]. Our results are consistent with previous data, suggesting that in T-LGLL additional somatic mutations can arise in the pre-expanded clonotypes [47].

In CD8+ T-LGLL, *STAT3* mutations have been associated with autoimmune manifestations such as rheumatoid arthritis and neutropenia [2, 5, 8, 48, 49]. In CD4+ T-LGLL, neutropenia is uncommon, and similarly, in our cohort *STAT5B* mutated patients had normal ANC levels but increased lymphocyte and LGL counts. Irrespective of the mutation status, the phenotype of the CD4+ T-LGLL clone was in most (75%) cases CD16−CD56+. Although CD16+CD56+ phenotype was detected in only four cases, all these had mutated *STAT5B*. CD56+ immunophenotype has also been associated with *STAT5B* mutations in CD8+ T-LGLL [9] in which the LGLs are usually CD56-.

TCRβ-seq has provided new insights into the nature of clonal expansions in CD8+ T-LGLL [14] but has not been performed in CD4+ T-LGLL earlier. In accordance with the previous flow cytometry-based data, in CD4+ LGLL the *TRBV06* family was enriched in the most hyper-expanded clones. Interestingly, 25% of T-LGLL clonotypes were also found among the non-expanded TCR repertoire in CD4+ T-LGLL patients and 27% were found in healthy individuals. This was not observed in the CD8+ T-LGLL disease, consistent with previous results [14]. Although our data was not HLA-matched, this could hint that the eliciting antigen in CD4+ T-LGLL could be commonly encountered. Further, in CD4+ T-LGLL also the non-leukemic T-cell compartment was significantly more clonal than in healthy controls. It has been shown previously, that CD4+ T cells in healthy controls are much richer in their diversity as compared to CD8+ T cells [50]. Hence, it was interesting to note that the overall clonality was higher in CD4+ T-LGLL compared to CD8+ T-LGLL, but this could also be due to the relatively small sample size. However, the eliciting antigen(s) remain unknown, as no exact matches were found for the known antigen-specific TCRs, which could be due to the small amount of known class II pMHC–TCR pairs and potential bias in the HLA genotypes of our samples. Additionally, since previous literature has indicated that a significant proportion of CD4+ T-LGLL patients has an underlying disease or malignancy [3], it cannot be excluded that the triggering event is abnormal antigen stimulation such as tumor antigen.

More research is warranted to clarify whether CD4+ T-LGLL is a true hematological malignancy or a reactive disorder of the immune system as clonal T cell proliferations and somatic mutations are also observed in healthy controls and non-malignant diseases [26, 51]. Following the initial event, activating *STAT5B* mutations may boost the aberrant proliferation and clonal persistence. Despite the STAT5B hyperactivation and the presence of multiple *STAT5B* mutations, the clinical course of CD4+ T-LGLL is indolent, and patients rarely have symptoms, differentiating it from CD8+ T-LGLL and other mature and immature CD4+ T-cell leukemias and lymphomas.

## Supplementary information

Supplementary legends_revised

Supplementary figures_revised

Supplementary Table 1

Supplementary Table 2_revised

Supplementary Table 3

Supplementary Table 4_revised

Supplementary Table 5

Supplementary Table 6

Supplementary Table 7

## Data Availability

TCRβ sequencing data is freely available at https://clients.adaptivebiotech.com/pub/bhattacharya-2022-BCJ (10.21417/DB2022BCJ).
