# Supplementary material for: Identification of novel STAT5B mutations and characterization of TCRβ signatures in CD4+ T-cell large granular lymphocyte leukemia
Source: Blood Cancer J. 2022 Feb 24;12(2):31. doi: 10.1038/s41408-022-00630-8 (PMC8873566; doi:10.1038/s41408-022-00630-8)
Supplement: Supplementary file 1 — Supplementary legends_revised [file 41408_2022_630_MOESM1_ESM.docx]

Identification of novel *STAT5B* mutations and characterization of TCRβ signatures in CD4+ T large granular lymphocyte leukemia

Dipabarna Bhattacharya^1,2^*, Antonella Teramo^3^*, Vanessa Rebecca Gasparini^3^*, Jani Huuhtanen^1,2,4*^, Daehong Kim^1,2^, Jason Theodoropoulos^1,2,4^, Gianluca Schiavoni^5^, Gregorio Barilà^3^, Cristina Vicenzetto^3^, Giulia Calabretto^3^, Monica Facco^3^, Toru Kawakami^6^, Hideyuki Nakazawa^6^, Brunangelo Falini^5^, Enrico Tiacci^5^, Fumihiro Ishida^7^, Gianpietro Semenzato^3^, Tiina Kelkka^1,2&^, Renato Zambello^3&^, Satu Mustjoki^1,2,8&$^

^1^ Hematology Research Unit Helsinki, University of Helsinki and Helsinki University Hospital Comprehensive Cancer Center, Helsinki, Finland;

^2^ Translational Immunology Research Program and Department of Clinical Chemistry and Hematology, University of Helsinki, Helsinki, Finland;

^3^ Department of Medicine, Hematology and Clinical Immunology Branch, University of Padova and Veneto Institute of Molecular Medicine (VIMM), Padova, Italy;

^4^ Department of Computer Science, Aalto University, Espoo, Finland;

^5^ Institute of Hematology and Center for Hemato-Oncology Research, University and Hospital of Perugia, Perugia, Italy;

^6^ Department of Internal Medicine, Division of Hematology, Shinshu University School of Medicine, Matsumoto, Japan;

^7^ Department of Biomedical Laboratory Sciences, Shinshu University School of Medicine, Matsumoto, Japan;

^8^ iCAN Digital Precision Cancer Medicine Flagship, Helsinki, Finland.

* Co-first authors

^$^ Corresponding author

^&^ Co-last authors

**SUPPLEMENTARY TABLES LEGEND**

**Supplementary Table 1: List of primers for *STAT5B* targeted sequencing.**

The table summarizes the nucleotide sequences and the genomic positions of each upstream/downstream locus-specific primer.

**Supplementary Table 2: List of primers for *STAT5B* mutagenesis.**

The table has all primers used in inducing nucleotide changes in the *STAT5B* gene to construct the 5 mutations studied in the paper.

**Supplementary Table 3: Clinical features of the 35 CD4+ T-LGLL patients.**

Patient specific clinical features are shown. F: female; M: male; WBC: white blood cells; LGL: large granular lymphocytes; ANC: absolute neutrophil count; Hb: hemoglobin; PLT: platelets; IF: immunophenotype; NA: not available.

**Supplementary Table 4: Expanded T cell clones in CD4+ T-LGLL.**

Table showing all clonal expansions >5% in the patient cohort considered as possible LGLL clones in this study. Clones with QG motifs and TRBV06 (from Vβ13 family) genes are in bold, whereas frequency of the clone is colored based on the size of the clone as reported on the right side of the table.

**Supplementary Table 5: Public versus private clones in CD4+ T-LGLL**

27% (16 out of 59) CD4+ T-LGLL specific clones were found in our healthy cohort of 37 patients.

**Supplementary Table 6: Vβ gene family usage in CD4+ T-LGLL**

TRBV06 family was found in 20% (12/59) followed by TRBV05 in 10% of the total number of identified leukemic clones.

**Supplementary Table 7: GLIPH results**

Two statistically significant amino acid motifs, SDP and SLRG, found from the 59 CD4+ T-LGLL clones.

# SUPPLEMENTARY FIGURE LEGENDS

**Supplementary Figure 1: Clonal expansions in CD4+ T-LGLL and comparison of the TCRβ repertoires between the CD4+ T-LGLL and healthy samples with varying thresholds.**

The non-leukemic clonality of CD4+ T-LGLL samples as compared to CD4+ healthy controls with varying thresholds for the putative T-LGLL clone. Thresholds ranged from 3% to 7%. P-values were calculated with a Bonferroni corrected Mann-Whitney test.

**Supplementary Figure 2: Publicness of the CD8+ T-LGLL clones.**

1. Leukemic CD8+ T-LGLL clonotypes are predominantly private to patients and rarely found in healthy counterparts. CD8+ T-LGLL leukemic clones are shown on the Y axis. HC is healthy control. Frequency of the clone is color coded based on the size of the clone as reported on the right side of the heatmap.
2. CD8+ T-LGLL clonotypes found in publicly available healthy cohort data (n=786)[^29^](https://paperpile.com/c/Et9AC2/uC5hP) where the colors refer to the size of the clonotype. Frequency of the clone is color coded based on the size of the clone as reported on the right side of the plot.

**Supplementary Figure 3:**

The generation probabilities for the 59 CD4+ T-LGLL clonotypes as calculated with OLGA as a function of how many times the clonotype was detected in a cohort of healthy MNC TCR repertoires (n=786)[^29^](https://paperpile.com/c/Et9AC2/uC5hP). A negative correlation between TCR generation probabilities and publicness of the cohort indicates that shared clonotypes are public probably due to biases in V(D)J recombination, where clonotypes with high generation probabilities are more commonly made. The R^2^ and the P-value were calculated with Pearson correlation.

**Supplementary Figure 4:**

Heat map showing the V-gene usage of CD4+ T-LGLL and healthy CD4+ sorted TCR-samples as fractions of the total repertoires. Preferential expression of *TRBV6-5* and *TRBV6-6* genes, both belonging to Vβ13.1 family in CD4+ T-LGLL patients’ TCRβ repertoire.

**Supplementary Figure 5:**

1. Antigen-specificities of the non-leukemic TCR repertoire from CD4+ T-LGLL patients matched against VDJdb[^24^](https://paperpile.com/c/Et9AC2/W9VvW), a database containing class II HLA pMHC-TCR pairings, resulted in multiple matches. The most common targets identified in the VDJdb for CD4+ T-LGLL were *Influenza A*, followed by *CMV*.
2. A representative network plot from a CD4+ T-LGLL patient showing which TCR clonotypes show structural similarities, where each node is a TCR and similar TCRs are connected via an edge. The CD4+ T-LGLL clone (pink) shares amino acid level similarities with multiple non-leukemic clones (green).

Supplementary methods:

*STAT5B* luciferase reporter assay

For three novel STAT5b variants (E433K, V712E, and P685R), expression constructs were generated as previously described in Andersson *et al*[*^4^*](https://paperpile.com/c/ztEu3k/eHJ9a). The activating N642H mutation and wild-type (wt) *STAT5B* constructs were used as controls[^4^](https://paperpile.com/c/ztEu3k/eHJ9a). Briefly, single nucleotide variants were introduced in pCMV6-XL6 plasmid containing wt *STAT5B* through site-directed mutagenesis (Geneart® Site-Directed Mutagenesis system, Invitrogen). Mutagenesis primers ordered from Sigma Aldrich are provided in **Supplementary Table 2**. HeLa cells were transfected with pCMV6-XL6 *STAT5B* plasmids and the pGL4.52 *STAT5B* luciferase reporter plasmid using the Fugene®HD transfection reagent. After 48 hours, One-Glo luciferase reagent was added, and luminescence intensities were measured using the PheraStar plate reader (BMG Labtech). Experiments were run in triplicates.

Western blot assay

STAT5b variants (E433K, V712E, and P685R) were generated following the site-directed mutagenesis protocol using the Phusion Site-Directed Mutagenesis Kit (Catalog Number F541) from Thermo Scientific using following manufacturers protocol. Mutagenesis primers are provided in **Supplementary Table 2**. HeLa cells, transfected as mentioned above, transiently carrying *STAT5B* mutations were serum starved for 6 hours. Cells were lysed using RIPA buffer including Halt^TM^ protease and phosphatase inhibitor (Thermo Fisher Scientific), and western blot assay was performed using Trans-blot Turbo system (Bio-Rad) as previously described[^26^](https://paperpile.com/c/ztEu3k/i3UW). Primary antibodies against phospho-STAT5, STAT5, c-MYC, BCL-2, Pim-1, and β-actin were purchased from Cell Signaling Technology. Anti-mouse IgG and anti-rabbit IgG were used as secondary antibodies (Cell signaling technology).
