# Supplementary material for: Identification of novel STAT5B mutations and characterization of TCRβ signatures in CD4+ T-cell large granular lymphocyte leukemia
Source: Blood Cancer J. 2022 Feb 24;12(2):31. doi: 10.1038/s41408-022-00630-8 (PMC8873566; doi:10.1038/s41408-022-00630-8)
Supplement: Supplementary file 2 — Supplementary figures_revised [file 41408_2022_630_MOESM2_ESM.pptx]

## Slide 1
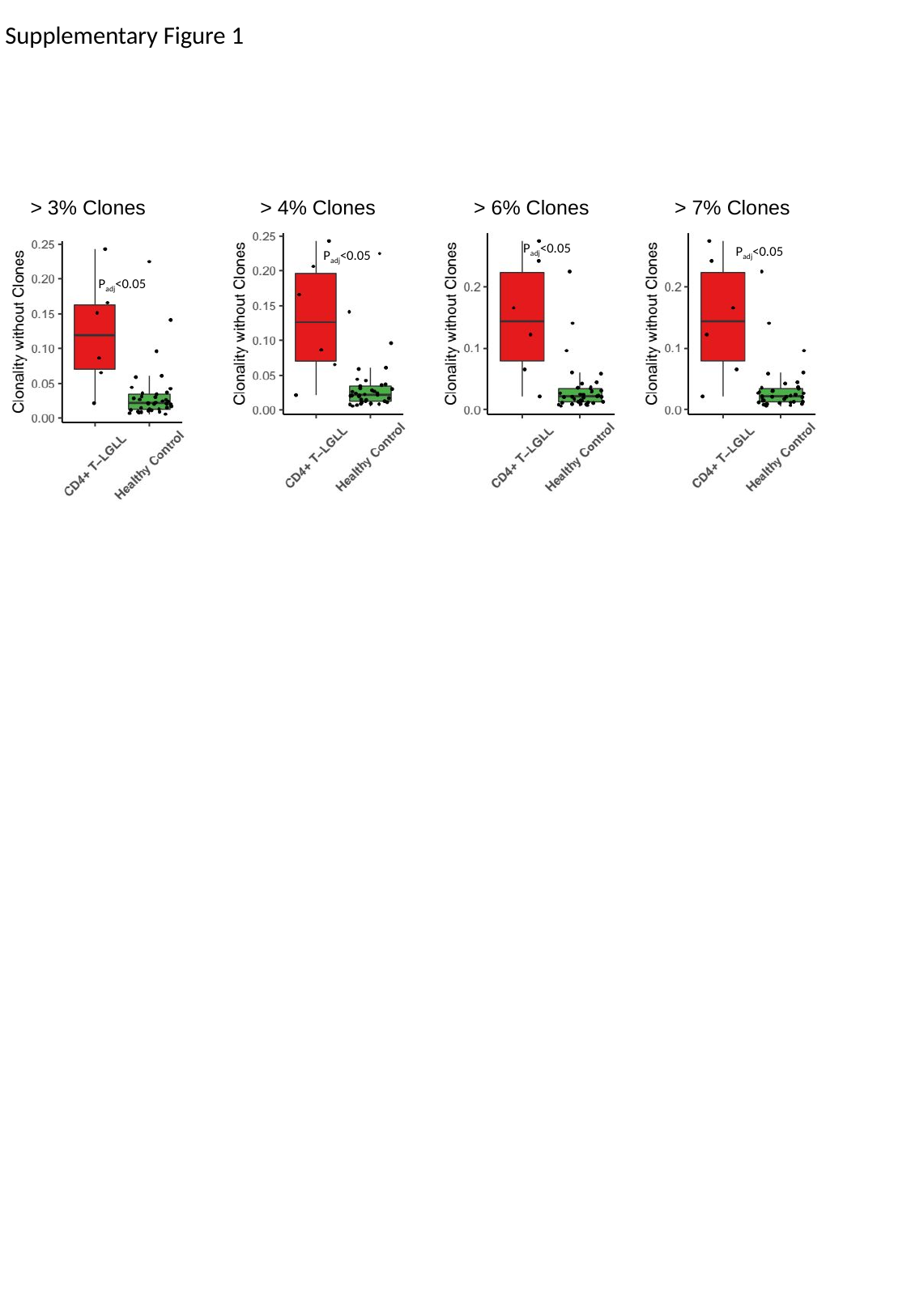

Supplementary Figure 1
> 7% Clones
> 4% Clones
> 6% Clones
> 3% Clones
Padj<0.05
Padj<0.05
Padj<0.05
Padj<0.05

## Slide 2
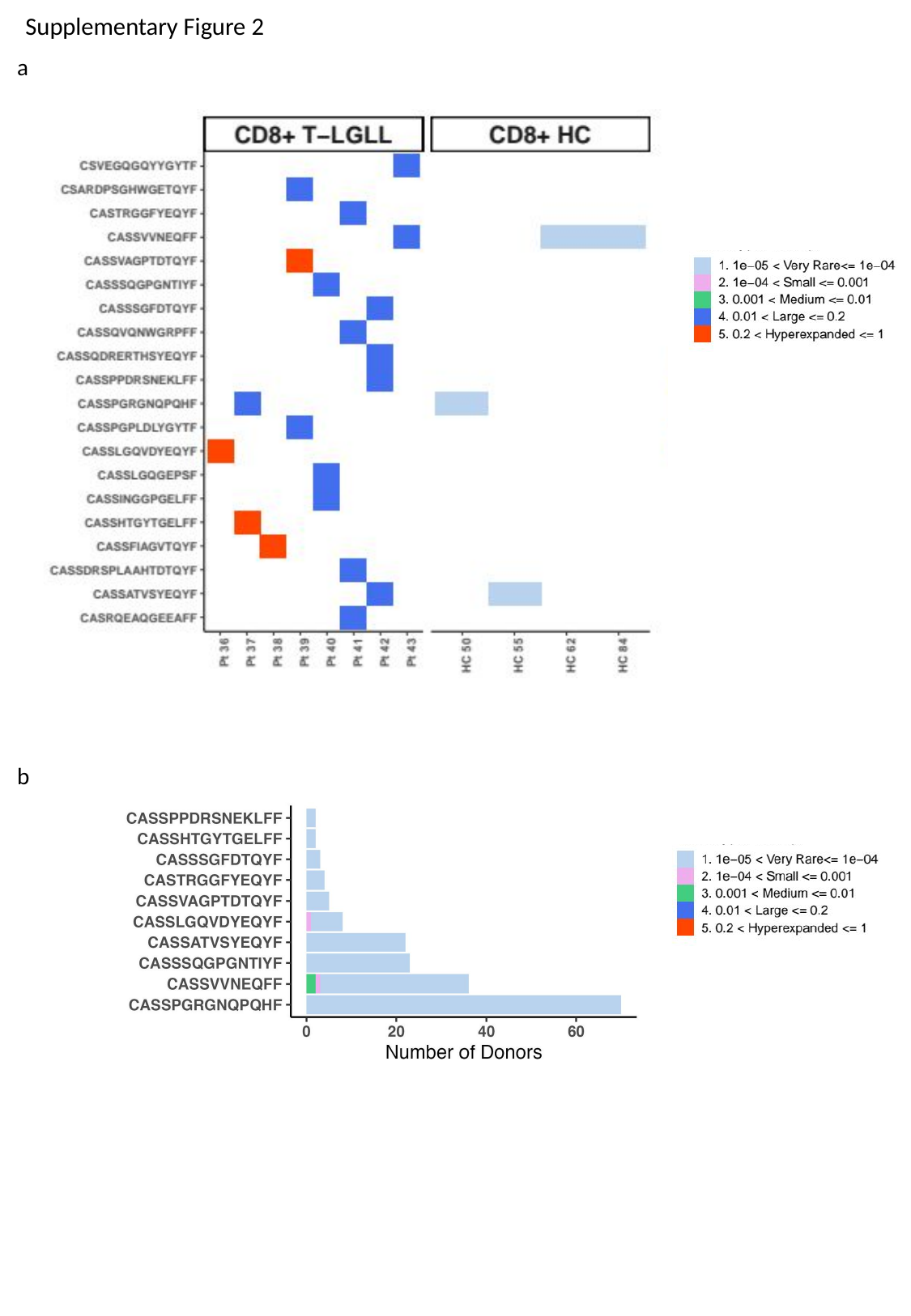

Supplementary Figure 2
a
b

## Slide 3
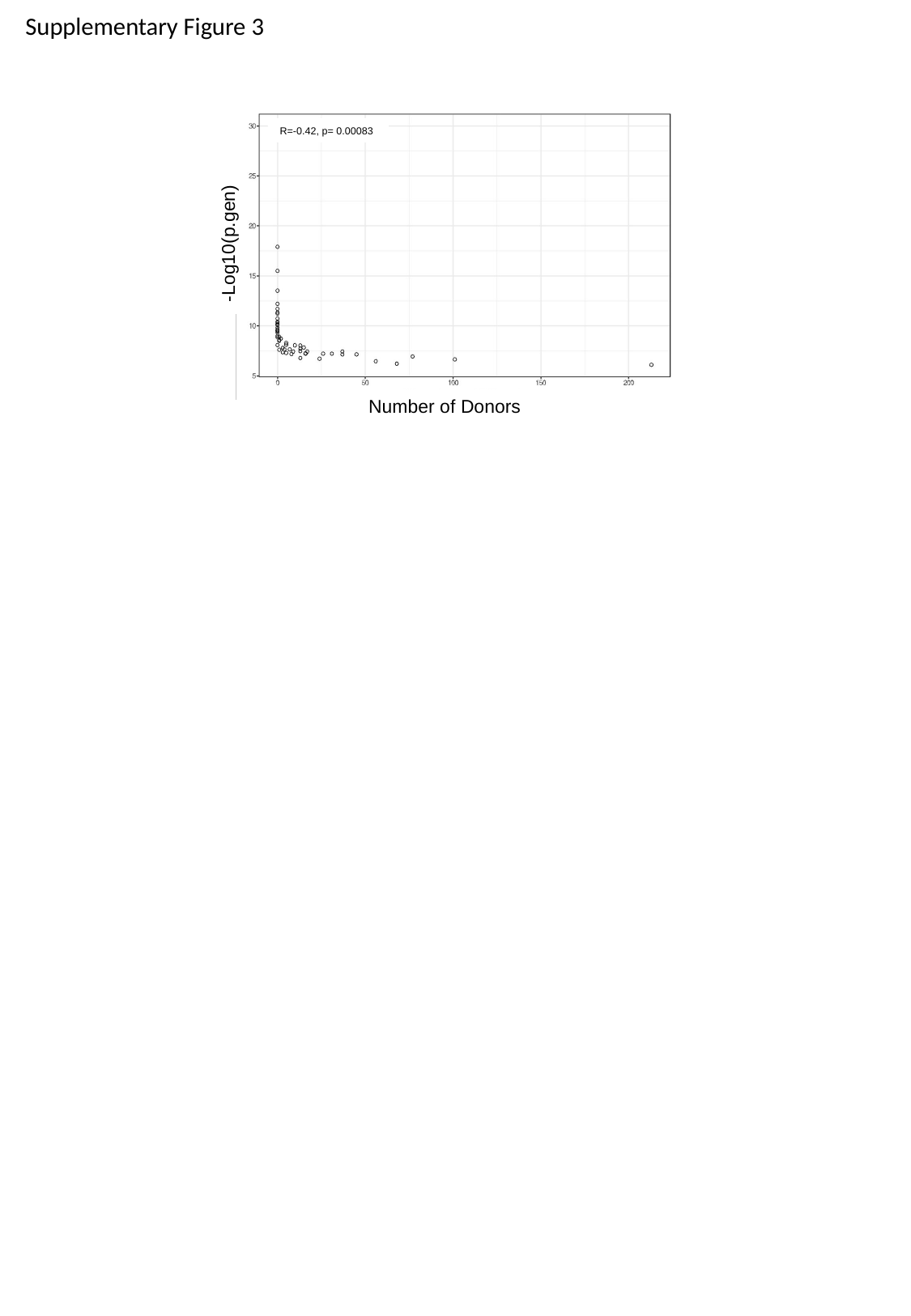

Supplementary Figure 3
R=-0.42, p= 0.00083
-Log10(p.gen)
Number of Donors

## Slide 4
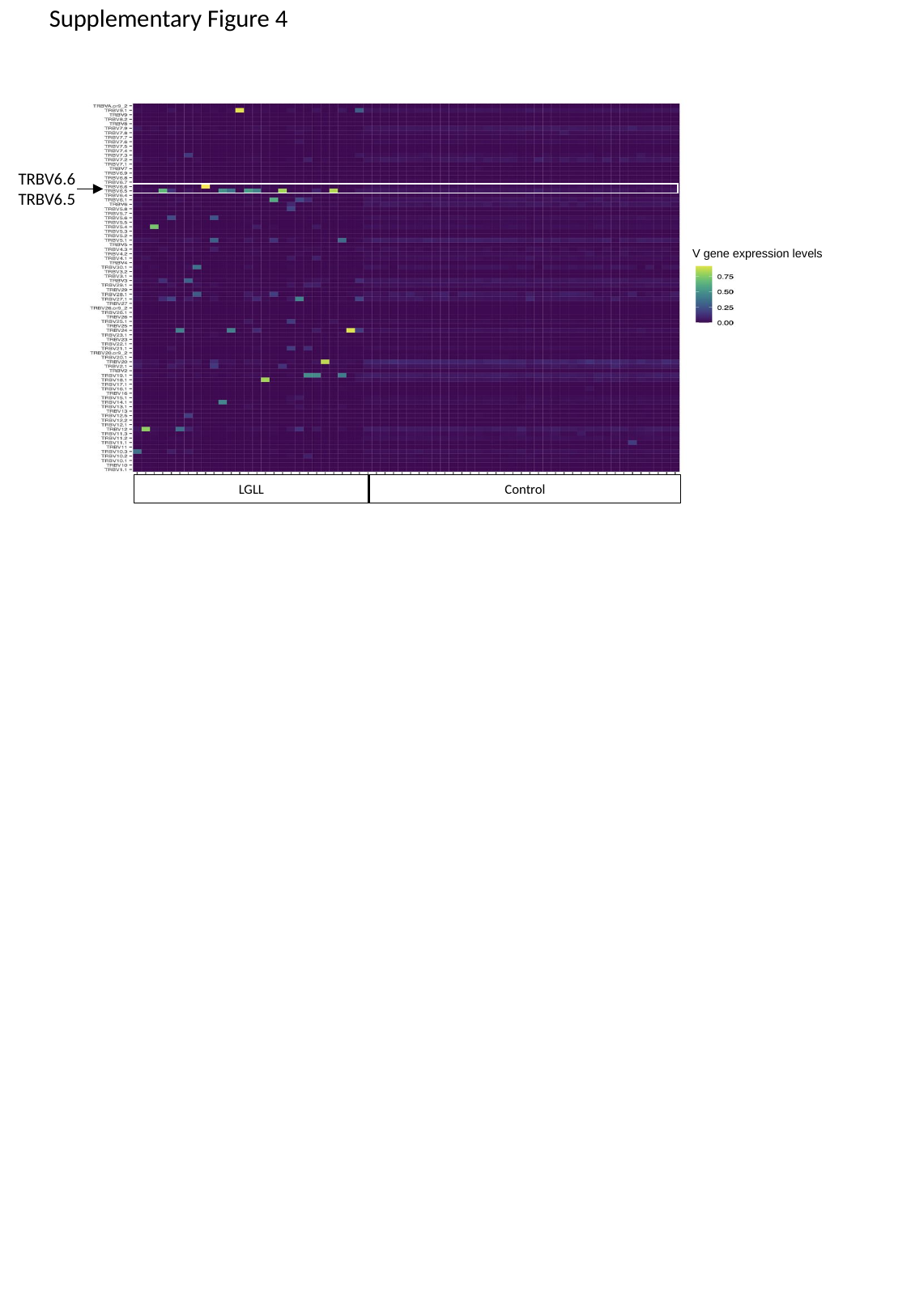

Supplementary Figure 4
LGLL
Control
TRBV6.6
TRBV6.5
V gene expression levels

## Slide 5
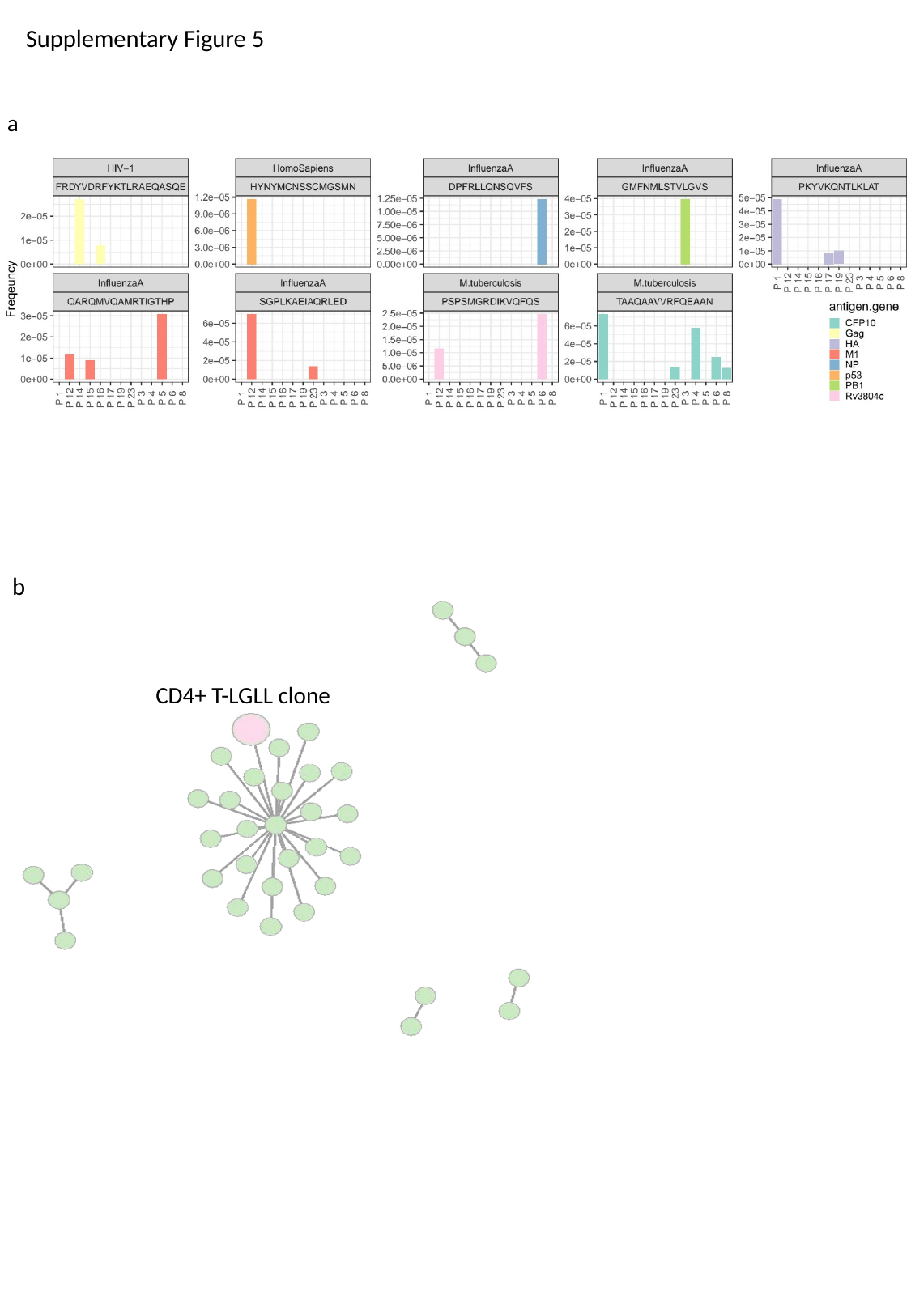

Supplementary Figure 5
a
b
CD4+ T-LGLL clone
